# Evaluation of iron overload: dual-energy computed tomography versus magnetic resonance imaging

**DOI:** 10.1186/1532-429X-16-S1-O92

**Published:** 2014-01-16

**Authors:** El-Sayed H Ibrahim, Andrew W Bowman

**Affiliations:** 1 Mayo Clinic, Jacksonville, Florida, USA

## Background

Iron toxicity is key factor for tissue damage in iron-overloaded patients, with induced heart failure as the main cause of death. T2*-weighted MRI has been established as the method of choice for evaluating iron-content with strong correlation with biopsy, where T2* < 20 ms and T2* < 10 ms at 1.5T indicate iron overload and severe iron overload, respectively. Recently-introduced dual-energy CT (DECT) has the potential for evaluating iron overload without energy-dependent CT attenuation or tissue fat effects. The aim of this study is to investigate the performance of DECT for iron mapping in in-vitro scans of calibrated iron phantoms and compare results to MRI.

## Methods

Ten 50-mL agarose phantoms(tubes) were created to mimic myocardial T1&T2 with different iron contents:0-4.5 g. The phantoms were imaged on Siemens 1.5T-MRI and DECT scanners. A GRE MRI sequence was used with 12-echoes (TE = 1-16 ms), 2.8 mm resolution, and 19 s scan-time. Two DECT scans were conducted with 80/140 kVp and 100/140 kVp, 0.3 mm resolution, and 2 s scan-time. 2-cm2 ROI's were used to measure average signal intensities in MRI and Hounsfield Units (HU) in CT. The values from MRI images were fitted to mono-exponential curves to measure T2* and R2* (= 1/T2*). Correlation analysis was conducted between iron-content, R2*, and HU values/differences/ratios at different energies (the 120 kVp-image automatically calculated by the scanner). Two tubes had T2* = 10 ms and 21 ms, which were used to identify boundaries between different iron-overload categories.

## Results

**Figure 1:**
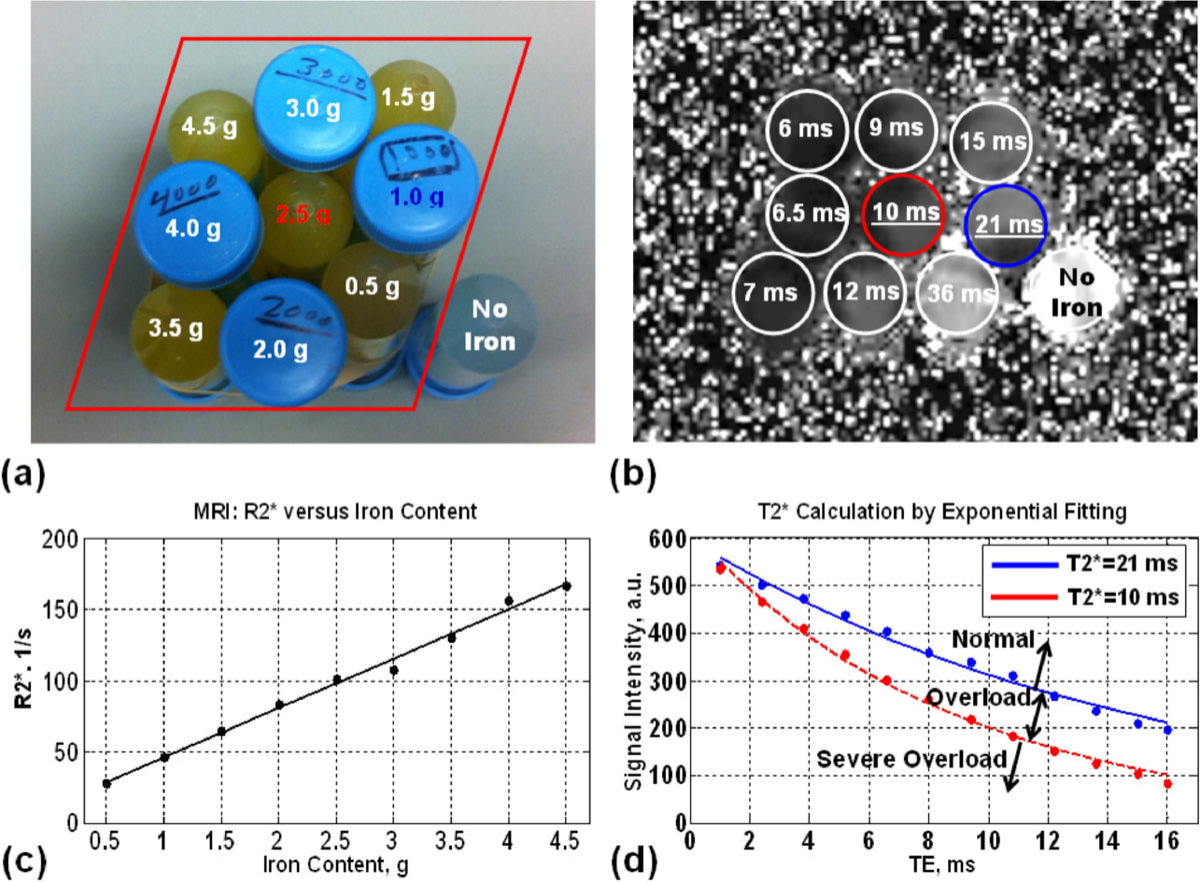
**MRI Results**. (a) Picture of the phantom with different iron contents (in grams) identified. (b) T2* map showing estimated T2* values for different tubes. (c) Linear relationship between iron content and R2* (= 1000/T2*). (d) Exponential fittings of the T2* relaxation curves versus echo time (TE) for the tubes that resulted in T2* of 21 ms (blue) and 10 ms (red), which represent the boundaries for iron overload and severe iron overload, respectively. Three regions can be identified on this figure for relaxation curves from tissues with normal iron content (T2* > 21 ms), iron overload (10 ms < T2* < 21 ms), and severe iron overload (T2* < 10 ms).

**Figure 2:**
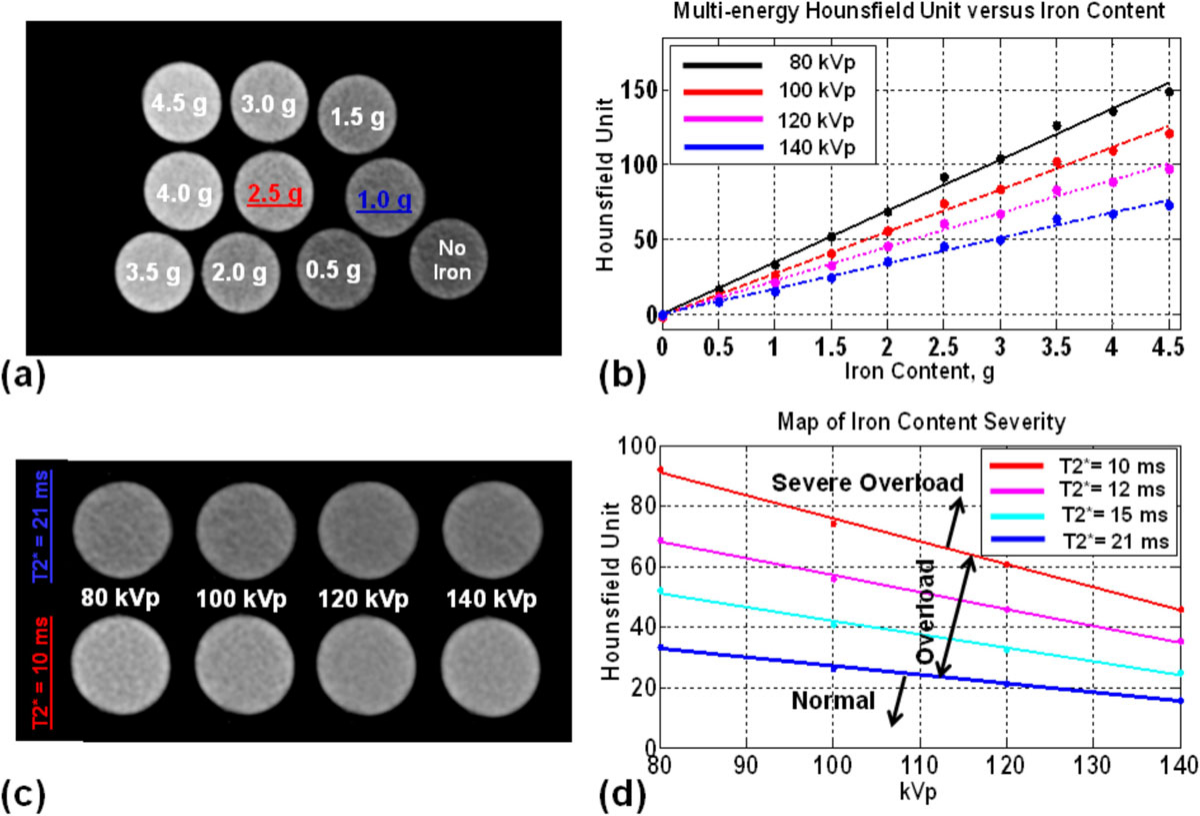
**Dual-energy CT Results**. (a) CT image of the phantom at one of the four acquired energies (100 kVp). (b) Relationships between Hounsfield unit and iron content at different energy levels (80, 100, 120, 140 kVp). (c) Sequence of CT images of the tubes that resulted in T2* values of 21 ms (top) and 10 ms (bottom), respectively, at different energy levels. (d) Linear relationships between Hounsfield unit and energy level for the iron tubes that resulted in T2* between 10 ms (red) and 21 ms (blue). The figure can be used as a map to identify the severity of iron overload based on the resulting Hounsfield unit and implemented kVp. Three regions can be identified on this map representing tissues with normal iron content (region below the blue line), iron overload (region between the blue and red lines), and severe iron overload (region above the red line).

Figures 1 &2 show the phantoms, T2*-map, CT images, plots of R2* and HU versus iron-content, T2* curves fitting, and DECT iron-map. T2* ranged from 6 ms to 36 ms. There were strong correlations between iron-content and R2*, HU values and HU differences at different energy levels (r > 0.95 and P < 0.0001). There were moderate correlations between iron-content and HU ratios (except 80/140) with 0.42 < |r| < 0.76 and 0.02 < P < 0.2. The T2* relaxation curves of the 21 ms and 10 ms tubes were used to differentiate between normal, overloaded, and severely-overloaded iron (Figure 1d). The HU values of these two tubes showed perfect linear relationships with energy-level (E) over diagnostic levels 80-140 kVp: HU = -0.29E+56.3 and HU = -0.76E+151.8 for the 21 ms and 10 ms T2*-values. The resulting CT-map (Figure 2d) is used to identify three regions: normal, overloaded, and severely-overloaded iron.

## Conclusions

DECT can be used for iron quantification with high accuracy similar to MRI, which might help in patient staging, independent of the energy level. New DECT scanners with low radiation-exposure, much shorter scan-time, and higher capability of measuring large iron-contents compared to MRI, may provide promising approach for evaluating myocardial iron overload.

## Funding

N/A.

